# Outcomes following percutaneous endoscopic gastrostomy versus fluoroscopic procedures in the Medicare population

**DOI:** 10.1016/j.sopen.2020.06.001

**Published:** 2020-07-17

**Authors:** Samantha Maasarani, Syed I. Khalid, Chantal Creighton, Athena J. Manatis-Lornell, Aaron L. Wiegmann, Samantha L. Terranella, Nicholas J. Skertich, Laura DeCesare, Edie Y. Chan

**Affiliations:** a Chicago Medical School, Rosalind Franklin University, Chicago, IL; b Rush Cook County Center for Outcomes Research and Department of Surgery, Rush University Medical Center, Chicago, IL; c Rush Medical College, Rush University Medical Center, Chicago, IL

## Abstract

**Background:**

In the United States, few high-quality manuscripts have directly compared the complication profiles of percutaneous endoscopic versus fluoroscopic gastrostomy. Thus, it is our goal to compare these 2 common procedures to better understand their efficacy and complication profiles.

**Materials and Methods:**

A retrospective analysis of patient records from Medicare parts A/B from 2007 to 2012 was used to identify percutaneous fluoroscopic gastrostomy and percutaneous endoscopic gastrostomy procedures. Patient demographics were stratified by age, sex, comorbidities, and complications.

**Results:**

A total of 258,641 patients were found to have either percutaneous fluoroscopic gastrostomy (26,477, 10.2%) or percutaneous endoscopic gastrostomy (232,164, 89.8%). Percutaneous fluoroscopic gastrostomy experienced greater rates for all complications queried. Multivariate analysis revealed that the percutaneous fluoroscopic gastrostomy cohort had statistically significant increased odds for short-term complications, such as ileus (odds ratio 1.4, 95% confidence interval 1.22–1.54), mechanical (odds ratio 2.4, 95% confidence interval 2.28–2.58), wound infection (odds ratio 1.4, 95% confidence interval 1.24–1.52), persistent fistula after tube removal (odds ratio 1.9, 95% confidence interval 1.78–2.12), and other complications (odds ratio 2.2, 95% confidence interval 2.03–2.37), and long-term complications, including abdominal wall pain (odds ratio 1.4, 95% confidence interval 1.33–1.44), wound infection (odds ratio 1.1, 95% confidence interval 1.01–1.15), and persistent fistula after tube removal (odds ratio 1.8, 95% confidence interval 1.72–1.87).

**Conclusion:**

Gastrostomy tubes are more frequently being placed via percutaneous endoscopic and fluoroscopic methods. This study suggests that those undergoing fluoroscopic placement have higher odds of developing short- and long-term postoperative complications.

## Highlights

• Fluoroscopic g-tubes have higher odds of developing short- and long-term complications.
• Fluoroscopy has higher odds for abdominal wall pain and mechanical complications.
• Hyperlipidemia, smoking, and hypertension are risk factors for abdominal wall pain.

## 1 INTRODUCTION

Gastrostomy tubes (g-tubes) are commonly placed in both adult and pediatric patients with several different conditions from neurologic, such as cerebral palsy and multiple sclerosis, to neoplastic, such as oropharyngeal and esophageal cancer [[1], [2], [3]]. G-tubes provide a means for patients to receive adequate nutrition, hydration, and medications via enteral administration [[4], [5], [6], [7], [8], [9], [10], [11], [12]]. There are several safe and widely accepted approaches for the insertion of g-tubes including open surgery, laparoscopic surgery, endoscopic placement, and fluoroscopic placement that are performed on both an inpatient and outpatient basis [[1], [2], [3],^11^].

Over the years, the methods of placement have evolved as a function of advancing technology and with the aim of improving outcomes and treatment efficiencies. The use of endoscopy versus laparoscopic and open procedures is largely dependent on a patient's specific needs [11,16,17]. Endoscopic placement is now the most commonly performed technique because it is less invasive, it is low cost, and there is no need for general anesthesia [18]. However, percutaneous endoscopic gastrostomy (PEG) and percutaneous fluoroscopic gastrostomy (PFG) have not been compared directly. Thus, it is our goal to compare these 2 common procedures to better understand their efficacy and complication profiles.

## 2 METHODS

A total of 51 million Medicare Standard Analytic Patient Records derived from Medicare parts A and B records from January 2004 to December 2014 were retrospectively analyzed. Patient undergoing endoscopic and fluoroscopic gastrostomy procedures were identified based on Current Procedural Terminology (CPT) codes and International Statistical Classification of Diseases (ICD)–9 diagnosis and procedure codes.

**Fig 1:**
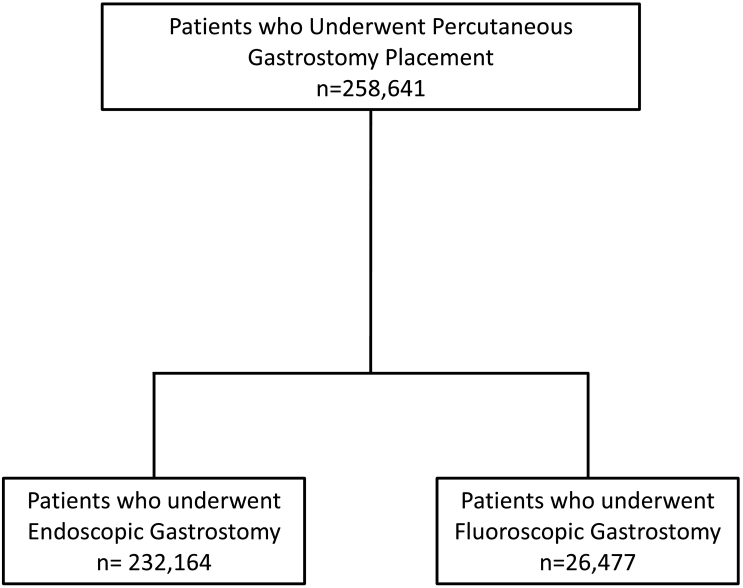
Patient selection flow diagram.

Patients undergoing gastrostomy procedures were identified by querying the database for incidence of CPT-43246 for PEG and CPT-39330 for PFG. Only patients undergoing endoscopic and fluoroscopic gastrostomy procedures were included in the study, with selection criteria summarized in Fig 1. The study was approved by the Institutional Review Board with a waiver of patient informed consent, as the nature of this analysis posed minimal risk to participating individuals, and the data were presented in aggregate to minimize any risks of loss of confidentiality of medical data. (See Table 1.)

**Table 1:** Descriptive characteristics for patients undergoing gastrostomy procedures

	*Total* n *= 258,641*	*Endoscopic* n *= 232,164*	*Fluoroscopic* n *= 26,477*	P *value*
Age (y), *n* (%)				
Unknown	2510 (0.97)	2270 (0.98)	240 (0.91)	<.05⁎
≤ 64	42,164 (16.3)	36,819 (15.9)	5345 (20.2)	<.05⁎
65–69	34,315 (13.3)	29,737 (12.8)	4578 (17.3)	<.05⁎
70–74	35,832 (13.9)	31,602 (13.6)	4230 (16.0)	<.05⁎
75–79	40,063 (15.5)	36,223 (15.6)	3840 (14.5)	.21
80–84	42,629 (16.5)	38,923 (16.8)	3706 (14.0)	<.05⁎
≥ 85	61,128 (23.6)	56,590 (24.4)	4538 (17.1)	<.05⁎
Sex, *n* (%)				
Male	125,349 (48.5)	110,933 (47.8)	14,416 (54.4)	<.05⁎
Female	130,781 (50.6)	118,961 (51.2)	11,820 (44.6)	<.05⁎
Unknown	2511 (0.97)	2270 (0.98)	241 (0.91)	<.05⁎
Comorbidities, *n* (%)				
Diabetes	99,781 (38.6)	89,650 (38.6)	10,131 (38.3)	.2684
Hypertension	189,501 (73.3)	169,026 (72.8)	20,475 (77.3)	<.05⁎
Hyperlipidemia	130,779 (50.6)	115,135 (49.6)	15,644 (59.1)	<.05⁎
Atrial fibrillation	59,659 (23.1)	53,346 (23.0)	6313 (23.8)	<.05⁎
Obesity	5975 (2.3)	5035 (2.2)	940 (3.6)	<.05⁎
Smoking	70,743 (27.4)	60,506 (26.1)	10,237 (38.7)	<.05⁎

⁎ Significant values *P* < .05.

### 2.1 Comorbidities

Demographic data for records included age and sex. ICD-9 diagnosis codes were used to identify comorbidities as previously described and listed in Supplementary Table 1. The following comorbidities were included in our study: diabetes mellitus, hypertension, hyperlipidemia, atrial fibrillation, obesity, and current or past smoking history.

### 2.2 Complications

Cohorts were queried to identify patients with complications occurring within 30 days or 6 months postoperatively based on ICD-9 diagnosis codes as previously described and listed in Supplementary Table 2. Postoperative complications occurring within 30 days were as follows: ileus; esophageal and gastric perforation; damage to other intra-abdominal organs, such as the colon (bowel perforation or colostomy); mechanical complication, including tube dysfunction, inadvertent g-tube removal, leakage of gastric contents, peristomal leakage, and gastrostomy dysfunction; wound infection; necrotizing fasciitis; persistent gastric fistula following gastrostomy tube removal; hematoma; and other complications, including gastrostomy tract tumor seeding and herniation of the stomach through a gastrostomy tube site. The following postoperative complications occurring within 6 months were included in our study: ulceration, wound infection, persistent fistula after g-tube removal, hematoma, gastric outlet obstruction, colocutaneous fistula, and abdominal wall pain.

### 2.3 Statistical Analysis

Descriptive statistics were calculated for age, sex, comorbidities, and postoperative complications for each respective cohort. Odds ratios (ORs) were calculated to compare the outcomes of specific complications based on which procedure was performed. The data were analyzed using R statistical software (version 3.42, 2017, R Project, Vienna, Austria).

**Fig 2:**
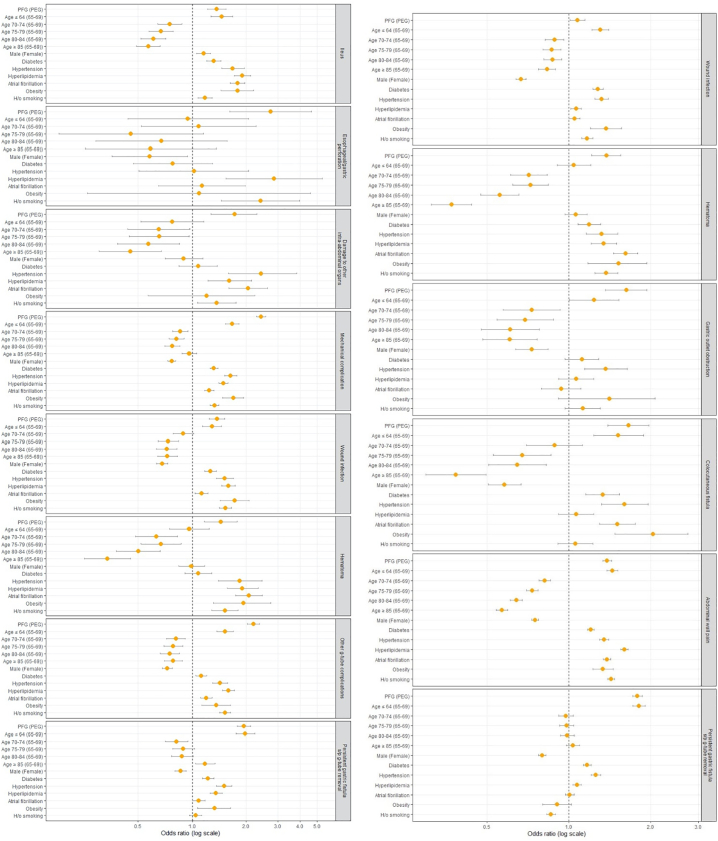
Multivariate regression models for short- and long-term complications occurring within 30 days (left) and 6 months (right) after percutaneous gastrostomy procedures.

Adjusted multivariate logistic regression models were constructed to identify associations between postoperative complications with age, sex, and comorbidities as seen in Fig 2 and Supplementary Tables 3 and 4. Models were constructed using *P* < .05 significance thresholds as demonstrated by univariate analysis and were adjusted for covariates of age, sex, and comorbidities. Continuous models using Hosmer and Lemeshow goodness-of-fit test and pseudo R2 measures were constructed for categorical outcomes using the method of McKelvey and Zavoina with both fixed and random effects, allowing estimates of percentage of variance explained by the model. All analyses met model assumptions and were constructed using Stata statistical software version 15 (StataCorp).

## 3 RESULTS

A total 258,641 patients undergoing percutaneous gastrostomy tube procedures met inclusion criteria; of these patients, 232,164 were placed endoscopically and 26,477 were placed fluoroscopically. Compared to the endoscopic cohort, those in the fluoroscopic group were significantly younger (*P* ≤ .01) and had greater rates of hypertension (77.3% vs 72.8%, *P* ≤ .01), hyperlipidemia (59.1% vs. 49.6, *P* ≤ .01), obesity (3.6% vs 2.2%, *P* ≤ .01), and smoking (38.7% vs 26.1%, *P* ≤ .01). No significant differences were found in the rates of diabetes or atrial fibrillation between the groups. The fluoroscopic cohort had greater rates of any complication (50.6% vs 43.9%), with the most common complications being abdominal wall pain (45.9% vs 41.4%), persistent fistula after g-tube removal (11.5% vs 7.1%), and mechanical (5.7% vs 2.4%) as seen in Table 2.

**Table 2:** Short-term and long-term complication rates

	*Total* n *= 258,641*	*Endoscopic* n *= 232,164*	*Fluoroscopic* n *= 26,477*	P *value*
Postoperative complications, *n* (%)				
Any complication	115,280 (44.6)	101,914 (43.9)	13,396 (50.6)	< .05⁎

Complications within 30 d postoperatively, *n* (%)				
Ileus	2217 (0.86)	1881 (0.81)	336 (1.3)	< .05⁎
Esophageal and gastric perforation	74 (0.03)	54 (0.02)	20 (0.08)	< .05⁎
Damage to other intra-abdominal organs	302 (0.12)	247 (0.11)	55 (0.21)	< .05⁎
Mechanical complication	6955 (2.7)	5459 (2.4)	1496 (5.7)	< .05⁎
Wound infection	3209 (1.2)	2734 (1.2)	475 (1.8)	< .05⁎
Necrotizing fasciitis	89 (0.03)	79 (0.03)	10 (0.04)	.89
Persistent fistula after g-tube removal	3654 (1.4)	3001 (1.3)	653 (2.5)	< .05⁎
Hematoma	633 (0.24)	528 (0.23)	105 (0.40)	< .05⁎
Other complications	4046 (1.6)	3199 (1.4)	847 (3.2)	< .05⁎

Complications within 6 mo postoperatively, *n* (%)				
Ulceration	542 (0.21)	484 (0.21)	58 (0.22)	.7749
Wound infection	10,425 (4.03)	9265 (3.99)	1160 (4.38)	< .05⁎
Persistent fistula after g-tube removal	19,442 (7.52)	16,393 (7.06)	3049 (11.5)	< .05⁎
Hematoma	1997 (0.77)	1693 (0.73)	304 (1.15)	< .05⁎
Gastric outlet obstruction	949 (0.37)	795 (0.34)	154 (0.58)	< .05⁎
Colocutaneous fistula	950 (0.37)	791 (0.34)	159 (0.6)	< .05⁎
Abdominal wall pain	108,296 (41.9)	96,154 (41.4)	12,142 (45.9)	< .05⁎

⁎ Significant values *P* < .05.

Odds of developing postoperative complications following fluoroscopic g-tube procedures were significantly greater among any complication (OR: 1.31, 95% confidence interval [CI] 1.28–1.34) queried as seen in Table 3. Among complications occurring within 30 days postoperatively, the odds of developing ileus (OR: 1.57, 95% CI 1.40–1.77), esophageal and gastric perforation (OR: 3.25, 95% CI 1.94–5.43), damage to other intra-abdominal organs (OR: 1.95, 95% CI 1.46–2.62), mechanical complication (OR: 2.49, 95% CI 2.35–2.64), wound infection (OR: 1.53 95% CI 1.39–1.68), persistent fistula following tube removal (OR: 1.93, 95% CI 1.77–2.10), hematoma (OR: 1.75, 95% CI 1.42–2.15), and other gastrostomy complications (OR: 2.37, 95% CI 2.19–2.55) were significantly higher in patients undergoing fluoroscopic placement of g-tubes. Likewise, patients undergoing fluoroscopic placement of g-tubes were at increased odds of developing abdominal wall pain (OR: 1.20, 95% CI 1.17–1.23), colocutaneous fistulas (OR: 1.77, 95% CI 1.49–2.10), gastric outlet obstructions (OR: 1.70, 95% CI 1.43–2.02), wound infection (OR: 1.10, 95% CI 1.04–1.17), persistent fistula following tube removal (OR: 1.71, 95% CI 1.64–1.78), and hematoma (OR: 1.58, 95% CI 1.40–1.79) within 6 months postoperatively. No significant differences were found in odds of developing postoperative necrotizing fasciitis or ulceration between groups.

**Table 3:** Odds of postoperative complications occurring in fluoroscopic gastrostomy compared to endoscopic procedures

	*OR (95% CI)*
Any complication	1.31(1.28–1.34)
Complications within 30 d postoperatively	
Ileus	1.57 (1.40–1.77)
Esophageal and gastric perforation	3.25 (1.94–5.43)
Damage to other intra-abdominal organs	1.95 (1.46–2.62)
Mechanical complication	2.49 (2.35–2.64)
Wound infection	1.53 (1.39–1.68)
Necrotizing fasciitis	1.11 (0.57–2.14)
Persistent fistula after g-tube removal	1.93 (1.77–2.10)
Hematoma	1.75 (1.42–2.15)
Other gastrostomy complications	2.37 (2.19–2.55)
Complications within 6 mo postoperatively	
Ulceration	1.05 (0.80–1.38)
Wound infection	1.10 (1.04–1.17)
Persistent fistula after g-tube removal	1.71 (1.64–1.78)
Hematoma	1.58 (1.40–1.79)
Gastric outlet obstruction	1.70 (1.43–2.02)
Colocutaneous fistula	1.77 (1.49–2.10)
Abdominal wall pain	1.20 (1.17–1.23)

Primary outcomes of multivariate regression models revealed that PFG had statistically significant increased odds for every complication included in our study. Of the short-term complications, PFG was associated with the highest risk for developing mechanical issues (OR: 2.4, 95% CI 2.28–2.58), other complications (OR: 2.2, 95% CI 2.03–2.37), persistent fistula after removal (OR: 1.9, 95% CI 1.78–2.12), wound infection (OR: 1.4, 95% CI 1.24–1.52), and ileus (OR: 1.4, 95% CI 1.22–1.54) within 30 postoperative days (Fig 2, Supplementary Tables 3 and 4). Patients in the fluoroscopic cohort were also at higher odds of developing long-term complications, such as persistent fistula after removal (OR: 1.8, 95% CI 1.72–1.87), abdominal wall pain (OR: 1.4, 95% CI 1.33–1.44), and wound infection (OR: 1.1, 95% CI 1.01–1.15), compared to endoscopic gastrostomy (Fig 2, Supplementary Tables 5 and 6).

Secondary outcome analysis revealed that short-term development of ileus, mechanical complications, wound infection, and other complications are more likely to occur in patients aged 64 years or younger with a history of diabetes, hypertension, atrial fibrillation, obesity, and smoking (Supplementary Tables 3 and 4). Short-term development of persistent fistula after g-tube removal was more likely in patients younger than 64 with a history of diabetes, hypertension, hyperlipidemia, or obesity (Supplementary Table 4), whereas fistula development within 6 months postoperatively was more likely if there was a history of diabetes, hypertension, or hyperlipidemia (Supplementary Table 5). Other long-term complications, such as wound infection and abdominal wall pain, had risk factors including age 64 or younger or a history of any comorbidity included in our study (Supplementary Tables 5 and 6).

## 4 DISCUSSION

Gastrostomy tube placement has progressed from being done as an open procedure to laparoscopic to now most commonly endoscopic. Fluoroscopic-assisted gastrostomy tubes have become increasingly used over the years and have been traditionally associated with low rates of complications [19,20]. Given the rise of fluoroscopic tube placement, we sought to evaluate and compare short-term and long-term outcome of PFGs and PEGs.

We found that ileus, esophageal and gastric perforation, damage to other intra-abdominal organs, mechanical issues, persistent fistula, hematoma, and wound infection 30 days following surgery and gastric outlet obstruction, colocutaneous fistula, abdominal wall pain, hematoma, and wound infection 6 months following surgery are more common when g-tubes are placed fluoroscopically than endoscopically. Overall, those in the PFG cohort were more likely to have any complication occur, with the most common complications being abdominal wall pain, mechanical issues, and wound infection occurring within 6 months postoperatively.

There is conflicting evidence regarding if PFGs or PEGs are the safest approach. The results of our study contradict some prior literature on these procedures. Unlike our study, some found no significant difference for rates of postoperative ileus between groups [13,21]. Contrary to our results, prior literature reports no significant difference between PFG and PEG patient's odds of developing postoperative gastric outlet obstructions [8,17], wound infections [8,13,17], mechanical complications [8,11,[13], [14], [15],^21^,^22^], or gastrostomy tract seeding [17]. Other literature rates of postoperative pain range from 2.3% to 20% for PFG and 0% to 12% for PEG [8,10,17,19,23]. This is drastically different from our study, which resulted in 41.9% of PEG and 45.9% of PFG patients reporting abdominal wall pain. This difference may be explained by the time frame in which pain was measured, as prior studies have also found higher rates of pain in PFG patients as a late complication but no significant differences in the short term [17].

This is presently the largest study conducted to evaluate outcomes of PFG versus PEG placement, and it found significantly higher complication rates in the PFG cohort. Not only did this cohort have higher overall complications, but it also had significantly higher odds of developing nearly every short- and long-term postoperative complication in our analysis. However, the PFG cohort had significantly more patients younger than 65. A 2016 analysis of the Medicare population estimated that individuals younger than 65 accounted for 21.9% of Medicare spending despite only accounting for 15.4% of the beneficiary population [24]. Furthermore, the cost per beneficiary was $15,437 in those less than age 65 compared to $10,182 for individuals older than 65 [24]. This would suggest that younger individuals enrolled in Medicare have a higher medical expenditure due to having more severe disabilities or comorbidities than their older counterparts. This assumption is supported by several studies demonstrating that people with disabilities have consistently higher rates of comorbidities, such as obesity, smoking, diabetes, and cardiovascular disease [25,26].

Additionally, PFG patients had significantly more comorbidities, such as hypertension, hyperlipidemia, and smoking history. This intergroup variation in underlying comorbidities may have occurred for several reasons. As described above, a larger proportion of the PFG cohort was younger than 65 years old, which may infer that this group is in relatively poorer health than patients undergoing PEG. Second, this variation may be a result of the implications for each procedure. PEG can be performed in an ambulatory care setting with intravenous and local sedation and is contraindicated in severe coagulation disorders, hemodynamic instability, sepsis, severe ascites, peritonitis, abdominal wall infections at placement site, peritoneal carcinomatosis, gastric outlet obstructions, history of total gastrectomy, or prolonged ventilation assistance [18,27], whereas PFG placement has no absolute contraindications and can be performed using local anesthesia only [28]. Presumably, those undergoing fluoroscopic placement may have been unable to tolerate intravenous sedation or were poor candidates for endoscopy because of their underlying comorbidities.

Because of these contrasting patient characteristics, we also conducted a multivariable logistic analysis adjusting for significant variables on univariate analysis and still found that patients with PFGs had significantly higher odds of developing complications. Moreover, compared to other studies, the large sample size of our study further strengthens our findings.

Our study has several limitations that should be considered when interpreting its results. The identification of patient characteristics and outcomes relied on ICD-9 and ICD-10 codes, which may have affected our results because of potential underreporting, missed codes, and/or inaccurate codes. The accuracy of the data may be less reliable because it is not always specific and depends on subjective interpretation of ambiguous diagnostic codes in medical records. For example, there is no way to be certain if abdominal wall pain was a direct complication of the gastrostomy tube or if this was attributed to some unrelated gastrointestinal pathology (gallbladder disease, inflammatory bowel disease, gastroenteritis, etc). This certainly impacted why abdominal pain was the most commonly occurring complication in our study.

Despite these limitations, we hope providers find our study interesting and clinically relevant to their daily practice. More importantly, we hope surgeons will anticipate complications occurring when placing fluoroscopic gastrostomy tubes. Additionally, we hope that these findings spark interest leading to further investigation into the technique, management, and outcomes of patients undergoing percutaneous gastrostomy procedures.

In conclusion, gastrostomy tubes are more frequently being placed via percutaneous endoscopic and fluoroscopic methods. This study demonstrates that those undergoing fluoroscopic placement have higher odds of developing short- and long-term postoperative complications.

## Author Contribution

**Samantha Maasarani:** Conceptualization, Methodology, Formal analysis, Investigation, Data curation, Writing - original draft, Writing - review & editing, Visualization. **Syed I. Khalid:** Conceptualization, Methodology, Software, Formal analysis, Investigation, Data curation, Writing - original draft, Writing - review & editing, Visualization. **Chantal Creighton:** Writing - original draft. **Athena J. Manatis-Lornell:** Writing - original draft. **Aaron L. Wiegmann:** Writing - original draft. **Samantha L. Terranella:** Writing - original draft. **Nicholas J. Skertich:** Writing - original draft. **Laura DeCesare:** Writing - original draft. **Edie Y. Chan:** Supervision.

## Conflict of Interest

The authors report no conflict of infect concerning the materials or methods used in this study or the findings specified in this paper.

## Funding Sources

This research did not receive any specific grant from funding agencies in the public, commercial, or not-for-profit sectors.

## Appendix A Supplementary data

Supplementary tables Image 1
