# Supplementary material for: Outcomes following percutaneous endoscopic gastrostomy versus fluoroscopic procedures in the Medicare population
Source: Surg Open Sci. 2020 Jul 17;3:2–7. doi: 10.1016/j.sopen.2020.06.001 (PMC8076911; doi:10.1016/j.sopen.2020.06.001)
Supplement: Supplementary file 1 — Supplementary tables [file mmc1.docx]

**Supplemental Table 1. Comorbidities by International Statistical Classification of Diseases (ICD) – 9 Diagnosis Codes**

| **Diabetes Mellitus** | 25000, 25001, 25002, 25003, 25010, 25011, 25012, 25013, 25020, 25021, 25022, 25023, 25030, 25031, 25032, 25033, 25040, 25041, 25042, 25043, 25050, 25051, 25052, 25053, 25060, 25061, 25062, 25063, 25070, 25071, 25072, 25073, 25080, 25081, 25082, 25083, 25090, 25091, 25092, 25093 |
| --- | --- |
| **Hypertension** | 4010, 4011, 4019 |
| **Stroke** | V1254 |
| **Hyperlipidemia** | 2720, 2721, 2722, 2723, 2724 |
| **Atrial fibrillation** | 42731 |
| **Obesity** | V8535, V8536, V8537, V8538, V8539, V8541, V8542, V8543, V8544, V8545 |
| **Smoking** | 3051, V1582 |

**Supplemental Table 2. Complications by International Statistical Classification of Diseases (ICD) – 9 Diagnosis Codes**

| **Complication:** | **ICD-9 Code:** |
| --- | --- |
| **Ileus** | 5601 |
| **Esophageal and gastric perforation** | 5304 |
| **Damage to other intra-abdominal organs:** includes bowel perforation or colotomy | 56983 |
| **Mechanical complications:** includes tube dysfunction, inadvertent G-tube removal, leakage of gastric contents, peristomal leakage, gastrostomy dysfunction | 53642 |
| **Wound infection** | 53641 |
| **Necrotizing fasciitis** | 72886 |
| **Persistent gastric fistula following gastrostomy tube removal** | 5374 |
| **Hematoma** | 99811, 99812, 99813 |
| **Other complications:** includes PEG tract tumor seeding, and herniation of the stomach through a PEG tube site | 53649 |
| **Ulceration** | 53100, 53101, 53110, 53111, 53120, 53121, 53130, 53131 |
| **Gastric outlet obstruction** | 5370 |
| **Colocutaneous fistula** | 56981 |
| **Abdominal wall pain** | 78900, 78901, 78902, 78903, 78904, 78905, 78906, 78907, 78909 |

**Supplemental Table 3. Multivariate Regression Model for ileus, esophageal/gastric perforation, damage to other intra-abdominal organs, and mechanical complications occurring within 30 days postoperatively**

|  | **Ileus**  p <0.05  *OR (95% CI)* | **Perforation**  p=0.70  *OR (95% CI)* | **Other injury**  p=0.86  *OR (95% CI)* | **Mechanical**  p<0.05  *OR (95% CI)* |
| --- | --- | --- | --- | --- |
| **Procedure** | | | | |
| Fluoroscopic | 1.4 (1.22-1.54) | 2.7 (1.62-4.63) | 1.7 (1.28-2.32) | 2.4 (2.28-2.58) |
| **Age** | | | | |
| ≤ 64 | 1.5 (1.27-1.68) | 1.0 (0.44-2.06) | 0.8 (0.52-1.16) | 1.7 (1.53-1.82) |
| 65-69 | *Reference value* | | | |
| 70-74 | 0.8 (0.64-0.88) | 1.1 (0.52-2.27) | 0.7 (0.44-0.97) | 0.9 (0.78-0.95) |
| 75-79 | 0.7 (0.58-0.79) | 0.5 (0.18-1.15) | 0.7 (0.45-0.97) | 0.8 (0.74-0.90) |
| 80-84 | 0.6 (0.52-0.71) | 0.7 (0.29-1.57) | 0.6 (0.38-0.85) | 0.8 (0.70-0.85) |
| ≥ 85 | 0.6 (0.49-0.66) | 0.6 (0.25-1.36) | 0.5 (0.30-0.67) | 1.0 (0.88-1.05) |
| **Gender** | | | | |
| Male | 1.2 (1.06-1.26) | 0.6 (0.36-0.94) | 0.9 (0.71-1.14) | 0.8 (0.73-0.81) |
| Female | *Reference value* | | | |
| **Comorbidities** | | | | |
| Diabetes | 1.3 (1.20-1.44) | 0.8 (0.47-1.29) | 1.1 (0.85-1.37) | 1.3 (1.25-1.39) |
| Hypertension | 1.7 (1.46-1.95) | 1.0 (0.50-2.06) | 2.4 (1.56-3.75) | 1.6 (1.51-1.77) |
| Hyperlipidemia | 1.9 (1.71-2.12) | 2.9 (1.54-5.34) | 1.6 (1.22-2.13) | 1.5 (1.41-1.58) |
| Atrial fibrillation | 1.8 (1.63-1.97) | 1.1 (0.65-1.97) | 2.1 (1.61-2.63) | 1.2 (1.17-1.32) |
| Obesity | 1.8 (1.45-2.22) | 1.1 (0.26-4.58) | 1.2 (0.61-2.37) | 1.7 (1.48-1.94) |
| Smoking | 1.2 (1.07-1.29) | 2.4 (1.45-3.99) | 1.4 (1.07-1.76) | 1.3 (1.26-1.40) |

**Supplemental Table 4. Multivariate Regression Model for wound infection, persistent fistula after g-tube removal, hematoma, and other complications occurring within 30 days postoperatively**

|  | **Wound infection**  p<0.05  *OR (95% CI)* | **Persistent fistula**  p<0.05  *OR (95% CI)* | **Hematoma**  p=0.62  *OR (95% CI)* | **Other comp.**  p<0.05  *OR (95% CI)* |
| --- | --- | --- | --- | --- |
| **Procedure** | | | | |
| Fluoroscopic | 1.4 (1.24-1.52) | 1.9 (1.78-2.12) | 1.4 (1.16-1.78) | 2.2 (2.03-2.37) |
| **Age** | | | | |
| ≤ 64 | 1.3 (1.14-1.45) | 2.0 (1.75-2.23) | 1.0 (0.75-1.24) | 1.5 (1.37-1.69) |
| 65-69 | *Reference value* | | | |
| 70-74 | 0.9 (0.79-1.02) | 0.8 (0.71-0.95) | 0.6 (0.48-0.83) | 0.8 (0.72-0.92) |
| 75-79 | 0.7 (0.65-0.84) | 0.9 (0.78-1.03) | 0.7 (0.52-0.87) | 0.8 (0.70-0.89) |
| 80-84 | 0.7 (0.63-0.83) | 0.9 (0.77-1.01) | 0.5 (0.38-0.66) | 0.8 (0.67-0.85) |
| ≥ 85 | 0.7 (0.64-0.83) | 1.2 (1.04-1.34) | 0.3 (0.25-0.45) | 0.8 (0.70-0.88) |
| **Gender** | | | | |
| Male | 0.7 (0.63-0.73) | 0.9 (0.80-0.93) | 1.0 (0.84-1.17) | 0.7 (0.68-0.78) |
| Female | *Reference value* | | | |
| **Comorbidities** | | | | |
| Diabetes | 1.3 (1.17-1.36) | 1.2 (1.14-1.31) | 1.1 (0.91-1.28) | 1.1 (1.05-1.20) |
| Hypertension | 1.5 (1.36-1.70) | 1.5 (1.36-1.66) | 1.8 (1.39-2.44) | 1.4 (1.30-1.58) |
| Hyperlipidemia | 1.6 (1.46-1.73) | 1.4 (1.25-1.46) | 1.9 (1.56-2.33) | 1.6 (1.47-1.71) |
| Atrial fibrillation | 1.1 (1.03-1.22) | 1.1 (0.999-1.18) | 2.1 (1.75-2.46) | 1.2 (1.11-1.29) |
| Obesity | 1.7 (1.43-2.08) | 1.3 (1.07-1.64) | 1.9 (1.34-2.80) | 1.4 (1.13-1.64) |
| Smoking | 1.5 (1.42-1.65) | 1.1 (0.97-1.13) | 1.5 (1.29-1.81) | 1.5 (1.42-1.64) |

**Supplemental Table 5. Multivariate Regression Model for wound infection, persistent fistula after g-tube removal, and hematoma occurring within 6 months postoperatively**

|  | **Wound infection**  p<0.05  *OR (95% CI)* | **Persistent fistula**  p<0.05  *OR (95% CI)* | **Hematoma**  p=0.16  *OR (95% CI)* |
| --- | --- | --- | --- |
| **Procedure** | | | |
| Fluoroscopic | 1.1 (1.01-1.15) | 1.8 (1.72-1.87) | 1.4 (1.21-1.56) |
| **Age** | | | |
| ≤ 64 | 1.3 (1.22-1.40) | 1.8 (1.72-1.92) | 1.0 (0.91-1.21) |
| 65-69 | *Reference value* | | |
| 70-74 | 0.9 (0.82-0.95) | 1.0 (0.91-1.03) | 0.7 (0.61-0.83) |
| 75-79 | 0.9 (0.80-0.93) | 1.0 (0.91-1.02) | 0.7 (0.62-0.84) |
| 80-84 | 0.9 (0.80-0.93) | 1.0 (0.91-1.03) | 0.6 (0.47-0.65) |
| ≥ 85 | 0.8 (0.77-0.89) | 1.0 (0.96-1.07) | 0.4 (0.31-0.44) |
| **Gender** | | | |
| Male | 0.7 (0.64-0.70) | 0.8 (0.78-0.83) | 1.1 (0.97-1.17) |
| Female | *Reference value* | | |
| **Comorbidities** | | | |
| Diabetes | 1.3 (1.22-1.33) | 1.2 (1.13-1.20) | 1.2 (1.08-1.31) |
| Hypertension | 1.3 (1.25-1.40) | 1.3 (1.22-1.32) | 1.3 (1.16-1.52) |
| Hyperlipidemia | 1.1 (1.02-1.12) | 1.1 (1.05-1.12) | 1.4 (1.22-1.50) |
| Atrial fibrillation | 1.1 (1.00-1.10) | 1.0 (0.97-1.05) | 1.6 (1.47-1.80) |
| Obesity | 1.4 (1.22-1.58) | 0.9 (0.82-1.05) | 1.5 (1.18-1.95) |
| Smoking | 1.2 (1.13-1.24) | 0.9 (0.85-0.91) | 1.4 (1.25-1.52) |

**Supplemental Table 6. Multivariate Regression Model for gastric outlet obstruction, colocutaneous fistula, and abdominal wall pain occurring within 6 months postoperatively**

|  | **Gastric outlet**  p=0.82  *OR (95% CI)* | **Colocutaneous fistula**  p=0.22  *OR (95% CI)* | **Abdominal wall pain**  p<0.05  *OR (95% CI)* |
| --- | --- | --- | --- |
| **Procedure** | | | |
| Fluoroscopic | 1.6 (1.37-1.94) | 1.7 (1.4-1.98) | 1.4 (1.33-1.44) |
| **Age** | | | |
| ≤ 64 | 1.2 (1.00-1.53) | 1.5 (1.23-1.88) | 1.5 (1.38-1.51) |
| 65-69 | *Reference value* | | |
| 70-74 | 0.7 (0.57-0.93) | 0.9 (0.70-1.12) | 0.8 (0.78-0.86) |
| 75-79 | 0.7 (0.54-0.88) | 0.7 (0.53-0.86) | 0.7 (0.70-0.77) |
| 80-84 | 0.6 (0.48-0.78) | 0.7 (0.51-0.83) | 0.6 (0.61-0.67) |
| ≥ 85 | 0.6 (0.47-0.76) | 0.4 (0.30-0.50) | 0.6 (0.54-0.60) |
| **Gender** | | | |
| Male | 0.7 (0.64-0.84) | 0.6 (0.51-0.67) | 0.8 (0.73-0.77) |
| Female | *Reference value* | | |
| **Comorbidities** | | | |
| Diabetes | 1.1 (0.97-1.29) | 1.3 (1.16-1.54) | 1.2 (1.17-1.24) |
| Hypertension | 1.4 (1.14-1.64) | 1.6 (1.32-1.96) | 1.4 (1.30-1.40) |
| Hyperlipidemia | 1.1 (0.92-1.24) | 1.1 (0.92-1.24) | 1.6 (1.55-1.65) |
| Atrial fibrillation | 0.9 (0.80-1.11) | 1.5 (1.30-1.76) | 1.4 (1.34-1.43) |
| Obesity | 1.4 (0.94-2.12) | 2.0 (1.50-2.78) | 1.4 (1.23-1.46) |
| Smoking | 1.1 (0.97-1.31) | 1.1 (0.91-1.23) | 1.4 (1.39-1.48) |
